# Potential negative impact of reputed regulators’ decisions on the approval status of new cancer drugs in Latin American countries: A descriptive analysis

**DOI:** 10.1371/journal.pone.0254585

**Published:** 2021-07-13

**Authors:** Carlos E. Durán, Martín Cañás, Martín Urtasun, Monique Elseviers, Robert Vander Stichele, Thierry Christiaens

**Affiliations:** 1 Clinical Pharmacology Research Group, Department of Basic and Applied Medical Sciences, Ghent University, Ghent, Belgium; 2 Federación Médica de la Provincia de Buenos Aires, La Plata, Argentina; 3 Instituto de Ciencias de la Salud, Universidad Nacional Arturo Jauretche, Florencio Varela, Argentina; University of California Berkeley, UNITED STATES

## Abstract

**Background:**

Many new cancer drugs are being approved by reputed regulatory authorities without evidence of overall survival benefit, quality of life improvement, and often based on clinical trials at high risk of bias. In recent years, most Latin American (LA) countries have reformed their marketing authorization (MA) rules to directly accept or abbreviate the approval process in case of earlier authorization by the European Medicines Agency (EMA) and the US Food and Drug Administration, mainly. This study assessed the potential impact of decisions taken by EMA regarding the approval of new cancer drugs based on no evidence of overall survival or in potentially biased clinical trials in LA countries.

**Design:**

Descriptive analysis.

**Setting:**

Publicly accessible marketing authorization databases from LA regulators, European Public Assessment Report by EMA, and previous studies accessing EMA approvals of new cancer drugs 2009–2016.

**Main outcome and measures:**

Number of new cancer drugs approved by LA countries without evidence of overall survival (2009–2013), and without at least one clinical trial scored at low risk of bias, or with no trial supporting the marketing authorization at all (2014–2016).

**Results:**

Argentina, Brazil, Chile, Colombia, Ecuador, Panama and Peru have publicly accessible and trustful MA databases and were included. Of the 17 cancer drugs approved by EMA (2009–2013) without evidence of OS benefit after a postmarketing median time of 5.4 years, 6 LA regulators approved more than 70% of them. Of the 13 drugs approved by EMA (2014–2016), either without supporting trial or with no trial at low risk of bias, Brazil approved 11, Chile 10, Peru 10, Argentina 10, Colombia 9, Ecuador 9, and Panama 8.

**Conclusions:**

LA countries keep approving new cancer drugs often based on poorly performed clinical trials measuring surrogate endpoints. EMA and other reputed regulators must be aware that their regulatory decisions might directly influence decisions regarding MA, health budgets and patient’s care elsewhere.

## Introduction

Regulatory authorities play a critical role to assure the entrance of effective and safe therapeutic products into the market. By principle, a good regulatory decision to approve a new drug must be preceded by a full and critical appraisal of pivotal clinical trials measuring endpoints meaningful to patients. At the end, one would expect that regulators do not approve a drug, or do not maintain the marketing authorization (MA) when the supporting trials showed to be biased, the expected results over clinical endpoints were not reached [1, 2], or those were not studied.

Unfortunately, recent studies give another picture. Regulatory decisions taken by European Medicines Agency (EMA) and US Food and Drug Administration (FDA) to approve new cancer drugs are often based on studies with no or disappointing results on primary clinical endpoints [3–9], and on methodologically doubtful clinical trials [10–12]. Davis and col. (2017) [3] found that up to 57% of drugs’ indications approved by EMA did not have evidence of overall survival (OS) or quality of life (QoL) improvement at the moment of MA, both *the* relevant clinical endpoints in oncology. Two years later, Naci and col. (2019) [10] showed that nearly half of the clinical trials underpinning EMA’s approvals of new cancer drugs were judged at high risk of bias, meaning bias arising from randomization, on-going deviations from intended interventions, missing outcome data, measurement of the outcome, or selective report of results.

This is not only important for Europe and the US, but also for the rest of the world. Latin American (LA) and the Caribbean region account for 7.8% of the total new cancer diagnoses in the world, with an estimated increase of 66% by 2030 [13]. The region experienced a decade (2006–2015) of sustained economic growth, with an increased access to new oncologic therapies. During this period, most LA countries reformed their MA rules to allow quicker access to new oncological drugs by directly recognizing the MA, or abbreviating the authorization processes, in case of earlier authorization by reputed regulators, such as EMA, FDA and few others [14, 15].

In this study, we sought to document the potential impact of decisions taken by a reputed regulator over the MAs of new cancer drugs in LA countries. In particular, we focused on the arguable cancer drugs authorized by EMA between 2009 and 2016, and approved by LA regulators. Additionally, we measured the time differences between EMA first MA and the first approval dates by LA countries.

## Materials and methods

### List of new cancer drugs and selection of Latin American countries

We constructed a list of cancer drugs approved for new indications by EMA as reported by Davis and col. for the period from 2009 to 2013 [3] and by Naci and col. for the period 2014 to 2016 [10]. We collected for each drug in the list, information about the MA status in LA countries, including the date of the first ever MA issued. Data was collected by one author (CD) and two other authors (MA/MU), working independently with mutual check, during November and December 2019, by scanning the regulator’s web sites of every LA country.

Two explicit exclusion criteria were applied. First, all medicinal products approved initially for an indication other than cancer were excluded. Second, products first approved by EMA before 2009 were also excluded because for approvals before 2009 it was difficult to ascertain the actual first entry dates in LA’s MA databases.

We searched for MA databases in the official regulator’s websites of the 20 continental LA countries (Argentina, Belize, Brazil, Bolivia, Chile, Colombia, Costa Rica, Ecuador, El Salvador, Guatemala, Guyana, Honduras, Mexico, Nicaragua, Panama, Paraguay, Peru, Suriname, Uruguay, Venezuela). Caribbean countries were not included in the search. Only countries having a publicly accessible and trustful MA database were included in the analysis. The minimum requirement was the presence of the active ingredient name and the brand name, presentation, authorization holder and the date of the first MA of the drug in the country.

### Data analysis

The starting point of our analysis to detect arguable oncological drugs were the studies of Davis and col. (2017) [3] and Naci and col. (2019) [10]. Davis analyzed the availability of evidence on OS at MA time and later in the postmarketing period (median of 5.4 year’s follow-up) of new cancer drugs approved by EMA between 2009 and 2013 (from here on renamed as *Survival list*). Naci reported the results of a risk of bias assessment of the clinical trials supporting the approval of new cancer drugs by EMA between 2014–2016 (from here on renamed as *Bias list)*. For both lists, we searched for the first MA date in the European Public Assessment Report website from EMA, the approval status of the drug in the selected LA countries, and the date of the first MA issued by each of the LA regulators.

The oncologic medicinal products and the corresponding indications listed in the *Survival list* were split as having or not available information about OS endpoint, either in the pivotal or in the postmarketing clinical trials. When more than one trial was available, and in case the information among those were discrepant, the drug was classified as having OS information if at least one of the trials/indications reported such information. Medicinal products from the *Bias list* were also dichotomously split as having or not a clinical trial scored at low risk of bias. For clinical trials with different assessment results for the same drug, we assigned the low risk of bias category if at least one of the trials was evaluated as such.

The descriptive analysis included the number (percentage) of new cancer drugs approved by EMA that were also approved by LA countries, the number (percentage) of cancer drugs approved by LA regulators with at least one OS time gain reported, or one clinical trial at low risk of bias. Finally, we measured for every cancer drug approved by the selected LA countries the time lapse between EMA and LA first MA dates; EMA dates were taken as the index date and the time differences measured in months. Results are presented as median and interquartile range (percentile 25^th^- 75^th^).

## Results

### Selection of cancer drugs and LA countries

**Fig 1:**
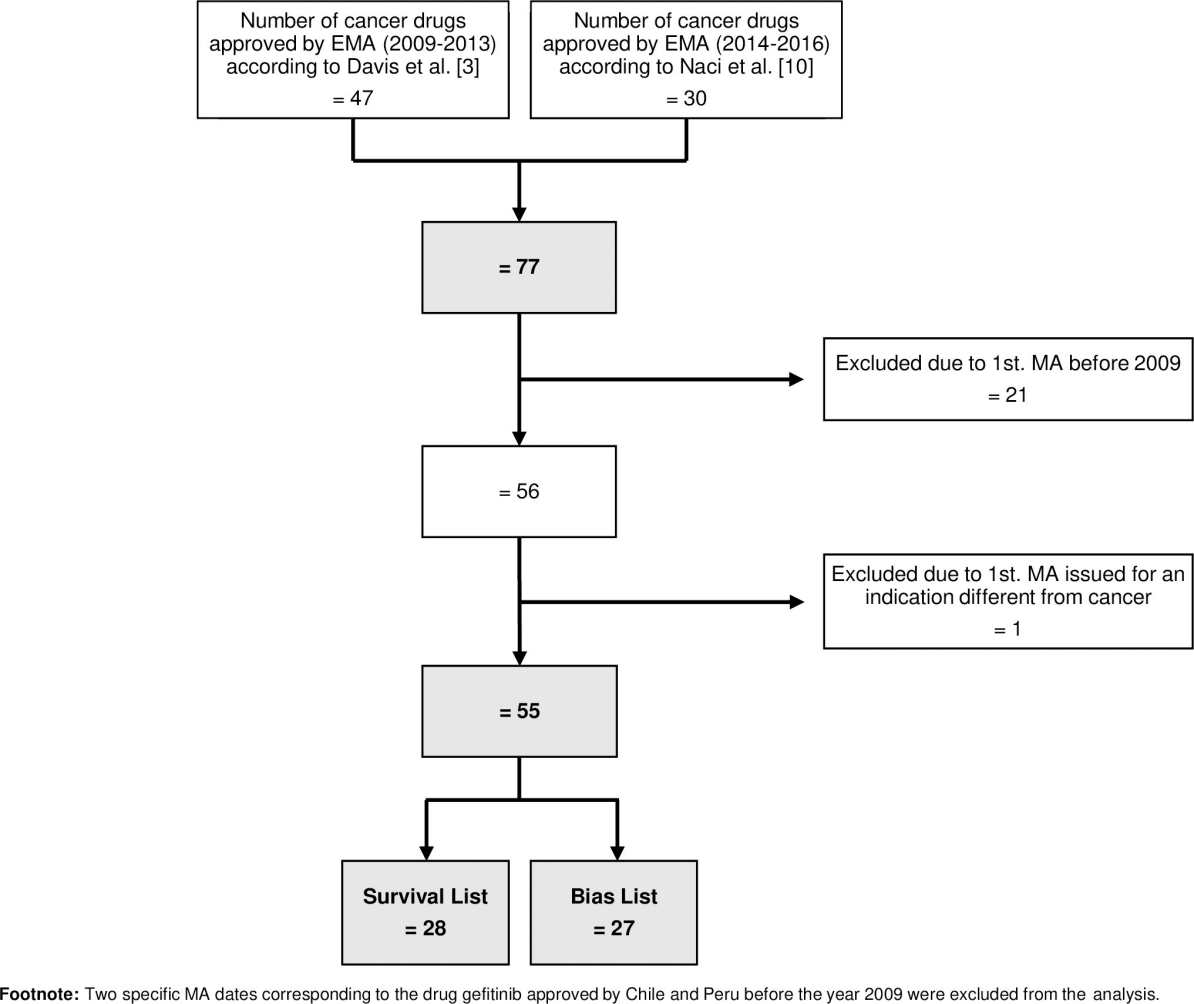
Selection of cancer drugs.

Fig 1 depicts the process to select the final list of oncologic medicinal products for this study. Of 77 new cancer drugs approved by EMA between 2009 and 2016, 22 were excluded; one was excluded because it was registered initially for a non-cancer indication (aflibercept), and 21 because the date of the first MA for a cancer indication by EMA was before the year 2009. Fifty-five new cancer drugs were finally included, 28 figuring on the Survival list, and 27 on the Bias list.

Only seven continental LA countries were finally selected (Argentina, Brazil, Chile, Colombia, Ecuador, Panama and Peru). These regulator´s websites provided public and trustful access to the required MA data, including information about current and former MA, names and dates. In the case of Mexico, we identified some major discrepancies about the date of MA among official sources. Belize, Guyana and Suriname are member States of the common Caribbean Regulatory System, and therefore were not included in the final analysis.

### Number of cancer drugs per country and time lapse between LA and EMA approvals

**Fig 2:**
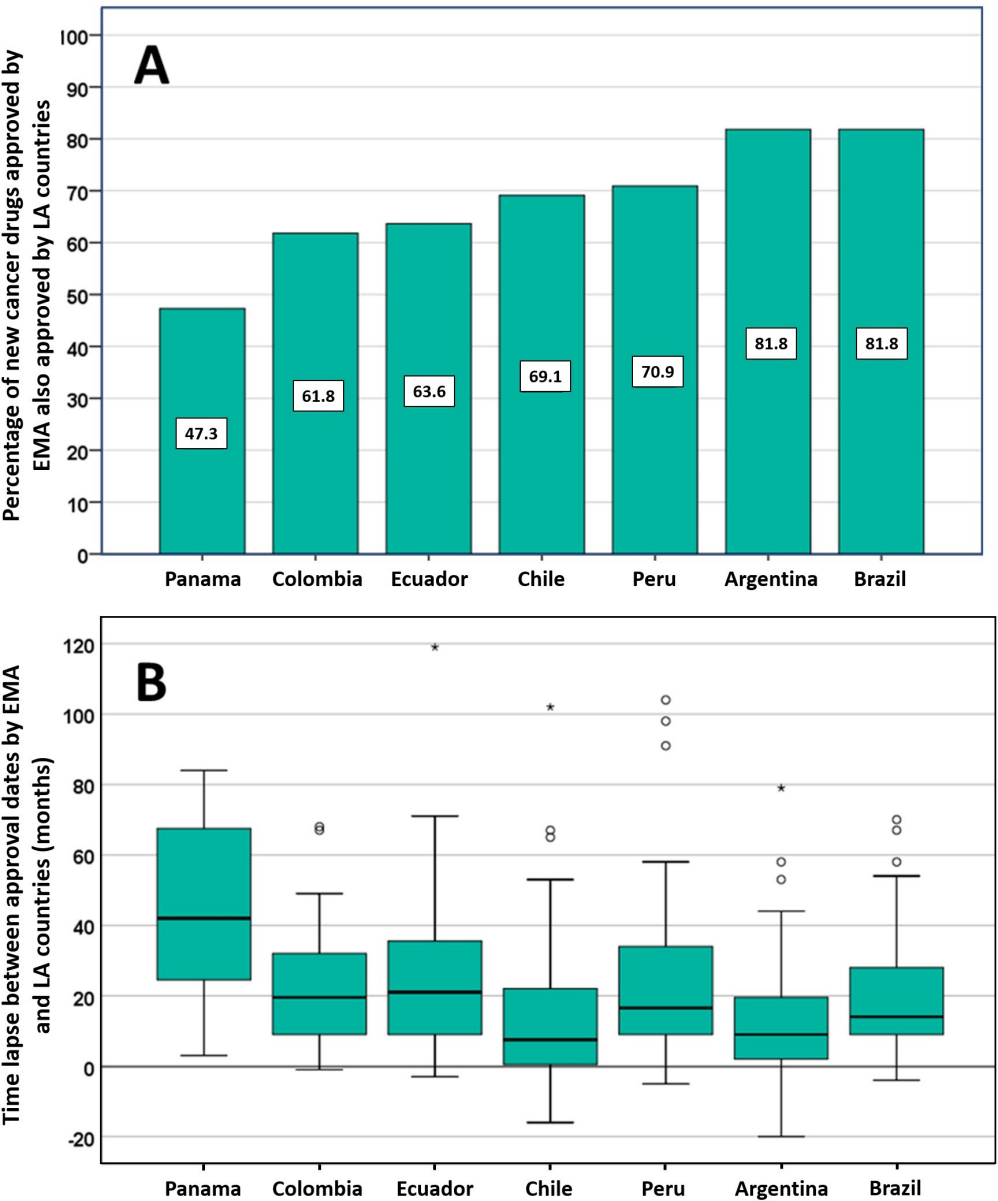
New cancer drugs approved by EMA (2009–2016), also approved by LA countries (panel A), and time lapse between LA countries’ and EMA’s first approval date (panel B).

Of the 55 new cancer drugs approved by EMA between 2009 and 2016, Panama approved 26 drugs (47.3%), Colombia 34 (61.8%), Ecuador 35 (63.8%), Chile 38 (69.1%), Peru 39 (70.9%), Argentina 45 (81.8%), and Brazil 45 (81.8%), see Fig 2, panel A. Five drugs (9.1%) were never approved by any of the 7 LA countries; in contrast, 19 drugs (34.5%) were approved by all of them.

Fig 2 panel B shows the median time [months—IQ range] between EMA MA and the first approval date by LA countries. With exception of Panama, the median approval time of LA countries after EMA first approval is less than two years, with Chile (7 months) and Argentina (9 months) being the quickest.

### Approvals of new cancer drugs by Latin American countries according to the overall survival benefit and the risk of bias assessment

**Fig 3:**
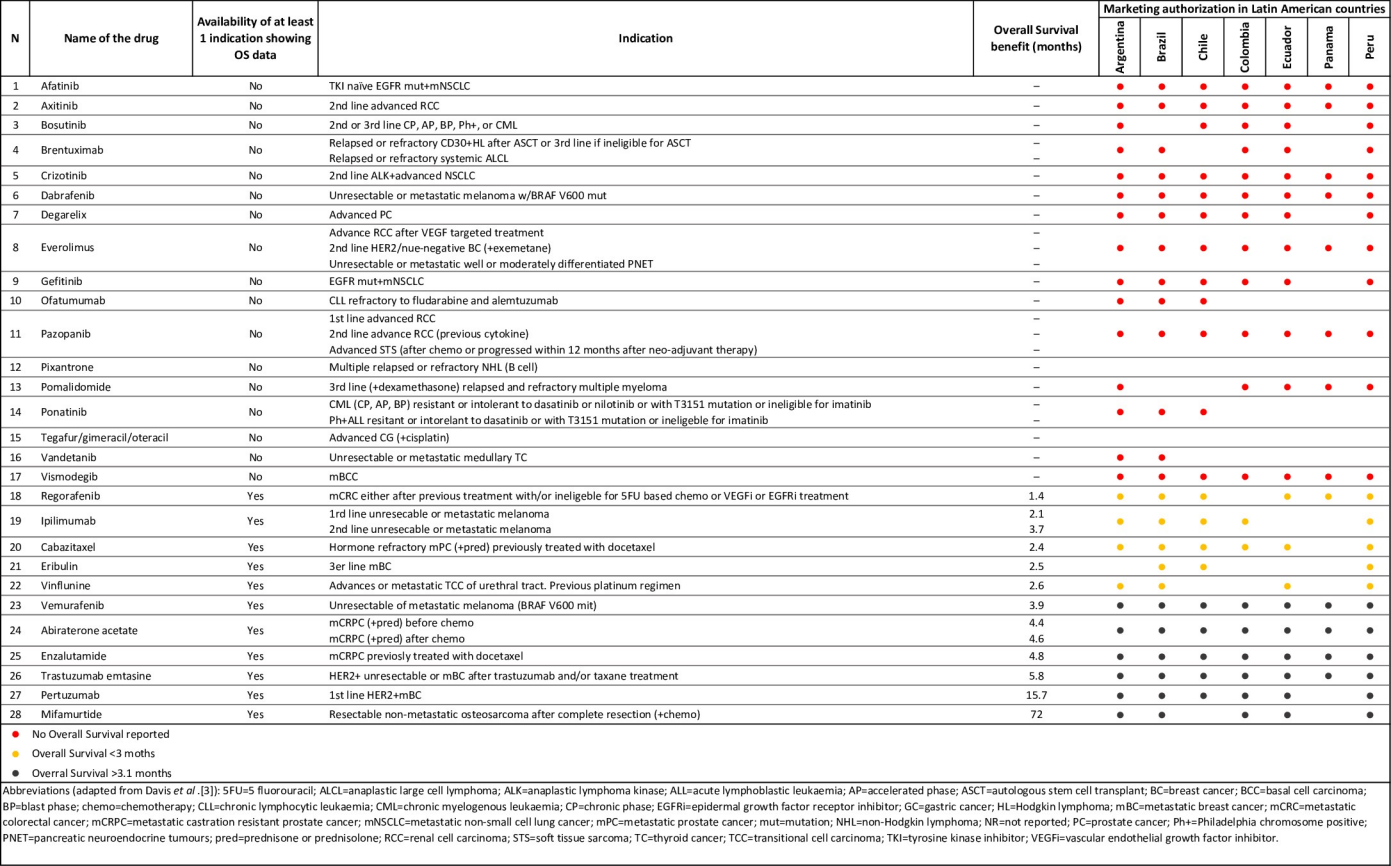
Overall survival benefit of new cancer drugs approved by 7 Latin American countries (2009–2013).

Fig 3 depicts the LA MA status of the drugs included in the Survival list; it shows the availability of OS endpoints over 28 drugs, and the corresponding MA in selected LA countries. Seventeen of 28 drugs did not report OS for the EMA approved indications at all, 6 reported OS gain for less than 3 months, and only 7 drugs showed an OS gain more than 3 months. Of the 17 drugs without any evidence of OS benefit, Argentina approved 15 (88.2%), Brazil 13 (76.5%), Chile 12 (70.6%), Colombia 12 (70.6%), Ecuador 12 (70.6%), Perú 12 (70.6%) and Panama 8 (47.1%), see Table 1.

**Table 1:** Summary of marketing authorizations per LA country according to the availability of OS data and risk of bias assessment of the cancer drugs approved by EMA between 2009–2016.

LA countries	Drugs included in the Survival list (n = 28)		Drugs included in the Bias list (n = 27)	
	No OS (n = 17)[100%]	OS reported (n = 11)[100%]	No trial or no trial at low risk of bias (n = 13)[100%]	At least one trial at low risk of bias (n = 14)[100%]
**Argentina**	15 [88.2]	10 [90.9]	10 [76.9]	11 [78.6]
**Brazil**	13 [76.5]	11 [100]	11 [84.6]	11 [78.6]
**Chile**	12 [70.6]	9 [81.8]	10 [76.9]	8 [57.1]
**Colombia**	12 [70.6]	8 [72.7]	9 [69.2]	6 [42.9]
**Ecuador**	12 [70.6]	9 [81.8]	9 [69.2]	6 [42.9]
**Panama**	8 [47.1]	5 [45.4]	8 [61.8]	6 [42.9]
**Peru**	12 [70.6]	11 [100]	10 [76.9]	7 [50.0]

**Fig 4:**
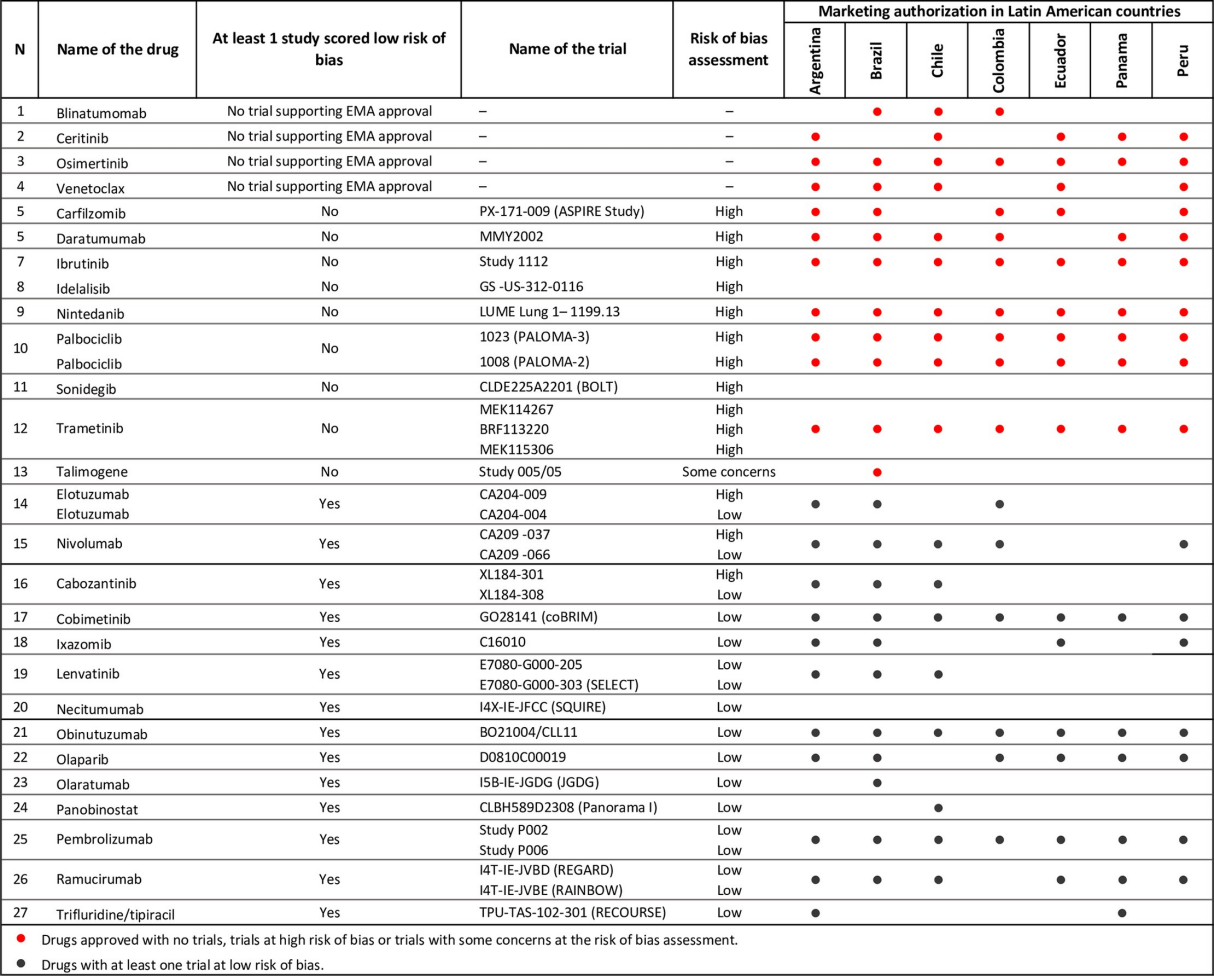
Clinical trials’ risk of bias assessment of new cancer drugs approved by 7 Latin American countries (2014–2016).

Fig 4 presents the LA MA status of the 27 drugs included in the Bias list. Fourteen of them showed at least one clinical trial scored at low risk of bias, 4 were approved without a trial supporting the dossier, and 9 without at least one trial scoring at low risk of bias. Of the 13 drugs approved either without supporting trial or with no trial at low risk of bias, Brazil approved 11 (84.6%), Chile 10 (76.9%), Peru 10 (76.9%), Argentina 10 (76.9%), Colombia 9 (69.2%), Ecuador 9 (69.2%), and Panama 8 (61.8%), see Table 1.

## Discussion

Our findings show that LA countries keep approving new cancer drugs mostly based on poorly performed clinical trials measuring surrogate endpoints. From the 17 cancer drugs approved by EMA between 2009 and 2013 without evidence of OS as primary endpoint, 6 LA regulators, except Panama, approved more than 70% of them. Similarly, from the 13 cancer drugs approved by EMA between 2014–2016 without at least one clinical trial scored at low risk of bias, or with no trial supporting the MA at all, LA regulators approved from 62% in Panama to 85% of them in Brazil.

The MA of arguable cancer drugs by LA countries might easily become the spearhead of several further issues, e.g. high prices of oncologic drugs unrelated to the LA countries´ minimum wage [16]; inequalities in the access to effective oncologic drugs [17]; reclamation of the drugs via the judicial system [18–21] and more, all leading to an uncontrolled rise in the expenditures of oncologic drugs. For instance, Brazilian public expenditure in antineoplastic drugs increased by 20 times between 2006 and 2013 [22], and the Ecuadorian public and private expenses in cancer drugs increased 22.6% annually between 2010 and 2014 [23]. Moreover, this is happening in health systems continuously facing financial constraints, fragmentation and inability to withdraw a drug when new contrasting evidence emerges.

During the last 3 decades, the idea that early access to new cancer drugs is always good for patients has become a global mantra [24, 25]. It has led to the relaxation of MA rules to quickly allow entrance of new cancer therapies into the worldwide market. Two main regulatory concepts have been used to facilitate this: *i*. the extended use of surrogate endpoints at registration time [26], and *ii*. the reliance on the regulatory decisions taken by reputed regulators [27–29].

First, surrogate endpoints are important in clinical research, mainly in early-phase clinical trials [30–32]. A surrogate endpoint is a biomarker that is intended to substitute for a clinical endpoint [31]. In oncology, common surrogate endpoints are Progression Free Survival (PFS), Disease Free Survival, Objective Response Rate, among few others [30, 32, 33]. The rationale behind the rise in the utilization of surrogate endpoints in oncology goes back to the mid-90’s, when President Clinton’s administration reformed the FDA’s approval standards in order to accept them for accelerated approval of new cancer drugs [34, 35]. The new policy established the foundations of measuring clinical endpoints mainly in postmarketing studies. However, these are seldomly performed nowadays [3, 36]. A central discussion regarding the use of clinical *versus* surrogate endpoints is that, notwithstanding surrogates intend to substitute clinical endpoints, several studies have shown that both are poorly correlated when OS or QoL are finally measured [3, 7, 37–40].

The clinical endpoints that really matter to cancer patients are OS and QoL. Ideally, these endpoints should be evaluated before the drug is approved by a regulator. Unfortunately, up to 57% of the drugs’ indications authorized by EMA between 2009 and 2013 came into the market without any evidence on OS or QoL [3], as has also been documented for the FDA [4]. Moreover, confirmatory trials reporting results from relevant clinical endpoints in the postmarketing period often fail to be performed. For instance, Davis et al. [3] proved that after a median post approval time of 5.4 years, 49% of the EMA approved drugs still did not show any significant improvement in OS or QoL. Similar results were reported by Kim et al. [7], where up to 57% of the cancer drugs approved by FDA based on surrogate endpoints failed to show OS benefit after a postmarketing period of 4.4 years.

Second, regulatory reliance has emerged as a broad concept where a national regulator relies on regulatory decisions taken by a third trusted party [27, 28, 41], usually a reputed regulator located in another jurisdiction. LA [15] as well as countries in other regions [42–45] have widely adopted it. Nowadays, several LA countries directly accept or abbreviate the MA process in case of earlier authorization by a designated trusted party, e.g. EMA, FDA and Health Canada are trusted regulators for all LA regulators relying on third parties. Hence, once a drug gets a MA issued by one of them, it becomes the golden key to be approved in 27 (out of 34) Latin American and Caribbean countries [15]. As example, of the 45 new drugs approved by Argentina in 2016, EMA and FDA earlier approved 78% and 80% of them, respectively. The Argentinian regulator applied the reliance rule in all cases [46]. A rather common argument in the field states that having approved a drug (based on a surrogate endpoint or not), not necessarily means that it will be prescribed or has to be covered by a public payer. However, in Latin American countries, where a strong private health sector is poorly regulated, once a drug achieves the market, pharmaceutical promotional activities are deployed to offer oncologist the new therapeutic options. Oncologic prescription patterns are highly influenced by the pharmaceutical industry, as has been described elsewhere [47–49]. This is followed by an increased pressure from medical associations, even patient´s organizations and the media to include the new drug into the lists of medicines to be publicly procured. Contrary to several European countries where the benefit and risk analysis is at the core of the decision making process by reimbursement agencies; economic analyses are not a common practice among decision bodies in LA countries. Hence, the MA of a new cancer drug by the country regulator is a critical step for LA health systems.

A key question remains however, how stringent is a regulator when half of the clinical trials supporting EMA regulatory decisions of new cancer drugs resulted to be highly risk of bias? [10]. In the same direction, Hilal T et al. [11] recently demonstrated that up to two thirds of the clinical trials leading to the approval of a new oncologic indication by the FDA had at least 1 limitation in a domain mining the quality of the trial. Moreover, it has been proven that the study design decreases in rigour when the drug is approved under special regulatory programs aimed to accelerate the MA [50, 51], as those implemented by EMA and FDA. By systematically approve new cancer drugs in less than two years (median time) after EMA approval (Fig 2 panel A), LA regulators are absorbing the strengths but also the weakness of the regulatory systems functioning under totally different contexts and motivations.

### Limitations

We focused our analysis on EMA’s approved products; this was used to illustrate the possible drawbacks in the process of relying on reputed regulators (e.g. EMA or FDA) by regulators that use to trust them. Within this framework, our study has several limitations. First, it was out of the scope of this study to search for new studies beyond the ones included in the Survival and Bias lists. It could be the case that, for some of them, new postmarketing trials would have changed the primary endpoint. However, as stated earlier in this discussion, postmarketing studies are rarely performed; so our main results will probably remain unchanged. Second, we did not have access to the full registration dossiers at included countries. Therefore, we do not know whether new studies were presented to LA regulators at registration time. Third, we could not provide information about how many drugs were actually presented by companies to every included country. The process of deciding when and where to present a registration dossier belongs entirely to the companies. As seen in our results, it could be that the larger the market is, the more drugs are presented for approval. To fully evaluate the regulatory performance of national regulators, it would be necessary to amend the lack of data on this subject.

### Policy implications

EMA and other reputed regulators must be aware that their regulatory decisions may directly impact downstream regulatory decisions in other countries and therefore, patients’ lives elsewhere. LA regulators must take into consideration that for new, questionable and expensive cancer drugs, reliance on marketing approvals by reputed regulators may boil down to signing a blank cheque. Therefore, the current MA rule allowing reliance on third parties should be re-evaluated to improve the process, while protecting patients and health systems’ resources; for instance, the practice of waiting for postmarketing results before granting a MA is a key point to consider for LA regulators and should become the rule, exceptionally broken under specific situations.

Speeding the access to essential drugs may be desirable. However, the governing axiom of early access to any new oncologic drug is jeopardizing the in-depth and careful evaluation of new medicines worldwide, with enormous consequences to low and middle income countries. Reliance may foster cooperation and resource optimization [52, 53] but it may also induce loss of national control over approvals of doubtful new medicines, and promote quick approvals where the capacity to monitor or withdraw MA when new contrasting evidence emerges is almost nonexistent [15].

## Conclusions

Six of seven LA countries approved more than 70% of the new cancer drugs authorized by EMA (2009–2013) without evidence of OS even after a median time of 5 years of MA. Similarly, from the 13 cancer drugs approved by EMA (2014–2016) without at least one clinical trial scored at low risk of bias, or with no trial supporting the MA at all, a large majority of them were approved by LA countries.

The European Medicines Agency and the US Food and Drug Administration must be aware that their regulatory decisions directly influence decisions elsewhere. The bar to approve new cancer drugs should be raised to protect patients globally [2, 26, 54]; this will require a paradigm shift from the industry driven early access approach to a new one prioritising the approval of clinically meaningful cancer drugs. A way to go further in that direction is by the regulators´ systematic demand of overall survival and quality of life, ideally both, as primary endpoints in oncologic clinical trials [1, 35, 54, 55]. This should be of particular interest for low and middle income countries, since early access to doubtful cancer drugs, approved on the basis of flawed evidence, could jeopardize countries´ health systems, the fair allocation of resources and services, and most importantly, result in suboptimal care to cancer patients.
